# Supplementary material for: Exclusive Breastfeeding Rates at Hospital Discharge Across the Robson Ten-Group Classification System: A Retrospective Study
Source: Nutrients. 2025 Nov 26;17(23):3708. doi: 10.3390/nu17233708 (PMC12694462; doi:10.3390/nu17233708)
Supplement: Supplementary file 1 [file nutrients-17-03708-s001.zip › nutrients-3922230-supplementary.pdf]

Table S1 Supplementary. Perinatal outcomes associated with the RTGCS and their relationship with EBF.

Robson group	*n*	Cs-rate (%)	Stillbirth (%)	χ^2 *p*-value	NICU admission (%)	χ^2 *p*-value	OR (CI95%) *p*-value	APGAR < 7 (%)	χ^2 *p*-value	OR (CI95%) *p*-value
1	6,845	11.852	0.4	356.628 <0.001	9.619	418.749 <0.001	NA	0.467	57.424 <0.001	NA
2	4,549	31.109	0.6		10.889		1.148 (1.015-1.299) 0.028	0.746		1.603 (0.987-2.601) 0.056
3	6,434	5.406	0.2		10.098		1.055 (0.942-1.183) 0.355	0.28		0.598 (0.335-1.066) 0.081
4	2,834	15.604	0.8		9.51		0.988 (0.851-1.146) 0.869	0.352		0.753 (0.370-1.534) 0.435
5	349	66.571	1.2		10		1.044 (0.730-1.494) 0.814	0		NA
6	520	98.088	2.5		17.208		1.953 (1.535-2.485) <0.001	0.382		0.818 (0.195-3.423) 0.783
7	219	96.818	2.8		16.818		1.900 (1.322-2.729) <0.001	0.455		0.973 (0.132-7.153) 0.979
8	238	62.762	1.7		25.941		3.291 (2.438-4.444) <0.001	0.418		0.895 (0.122-6.580) 0.972
9	63	100	5.2		17.188		1.950 (1.014-3.752) 0.045	4.688		10.480 (3.125-35.144) <0.001
10	986	25.823	6.8		28.614		3.766 (3.213-4.414) <0.001	1.496		3.235 (1.746-5.996) <0.001

NA: Not applied; CS: caesarean section; χ^2: Chi-squared test; OR: odds ratio; CI95%: Confidence Interval 95%, NICU: Neonatal Intensive Care Unit.

Note: Group 2 includes subgroups 2a and 2b; Group 4 includes subgroups 4a and 4b, according to the methodology for the exclusive breastfeeding (EBF) analysis.
